# Comparison of the Fatty Acid Metabolism Pathway in Pan-Renal Cell Carcinoma: Evidence from Bioinformatics

**DOI:** 10.1155/2021/8842105

**Published:** 2021-02-22

**Authors:** Ping Wu, Yingkun Xu, Jiayi Li, Xiaowei Li, Peizhi Zhang, Ningke Ruan, Cong Zhang, Panpan Sun, Qifei Wang, Guangzhen Wu

**Affiliations:** ^1^Department of Anesthesiology, The First Affiliated Hospital of Dalian Medical University, Dalian, China; ^2^Department of Urology, Shandong Provincial Hospital, Cheeloo College of Medicine, Shandong University, Jinan, China; ^3^School of Business, Hanyang University, Seoul, Republic of Korea; ^4^Department of Urology, The First Affiliated Hospital of Dalian Medical University, Dalian, China; ^5^The Nursing College of Zhengzhou University, Zhengzhou, China; ^6^Department of Pain Management, Shandong Provincial Hospital, Cheeloo College of Medicine, Shandong University, Jinan, China; ^7^Department of Pain Management, The Second Hospital of Shandong University, Jinan, China

## Abstract

This study analyzed and compared the potential role of fatty acid metabolism pathways in three subtypes of renal cell carcinoma. Biological pathways that were abnormally up- and downregulated were identified through gene set variation analysis in the subtypes. Abnormal downregulation of the fatty acid metabolism pathway occurred in all three renal cell carcinoma subtypes. Alteration of the fatty acid metabolism pathway was vital in the development of pan-renal cell carcinoma. Bioinformatics methods were used to obtain a panoramic view of copy number variation, single-nucleotide variation, mRNA expression, and the survival landscape of fatty acid metabolism pathway-related genes in pan-renal cell carcinoma. Most importantly, we used genes related to the fatty acid metabolism pathway to establish a prognostic-related risk model in the three subtypes of renal cell carcinoma. The data will be valuable for future clinical treatment and scientific research.

## 1. Introduction

Renal cell carcinoma (RCC) is one of the most common malignancies in the urinary system [1]. The incidence of kidney cancer has been increasing worldwide in recent years [2]. In the United States, nearly 64,000 patients were diagnosed as having RCC in 2017, with an annual increase of 2-4% [3, 4]. The primary treatment for RCC is radical resection of the kidney. However, there is no effective treatment for patients with metastatic and recurrent kidney cancer. Increasing evidence suggests that metabolic gene perturbations are a significant feature of RCC [5–7]. Epigenetic interference genes in RCC are good candidate targets for the development of powerful prognostic and diagnostic tools and novel therapies [8, 9]. The common pathological types of RCC mainly include kidney renal clear cell carcinoma (KIRC), kidney renal papillary cell carcinoma (KIRP), and kidney chromophobe (KICH).

It is unclear whether there are differences in fatty acid metabolism pathways between these three RCC subtypes. To explore this, we used gene set variation analysis (GSVA) to perform pathway analysis in pan-RCC. The approach sensitively detected subtle changes in pathway activity between tumor tissue and normal tissue [10].

The present data indicate that fatty acid metabolism pathways may be essential in the three RCC subtypes, with significant differences between subtypes. Metabolism is influential in RCC. Changes in fatty acid metabolism pathways may have an essential role in the development of RCC [11, 12]. A characteristic feature of cancer cells is that they can readjust their metabolism to maintain ATP production and the growth, division, and survival of the cancer cells. Notably, abnormally, fatty acid metabolism occurs in the development of RCC and in most tumors. The importance of abnormal changes in fatty acid metabolism in cancer has attracted the attention of many researchers, since these metabolites can be used as structural components of the cell membrane matrix and critical secondary messengers and as fuel sources for energy production [13–15].

In this study, to fully understand the genetic variation and clinicopathological correlation of these fatty acid metabolic genes in pan-RCC, we obtained a panorama of copy number variation (CNV), single-nucleotide variation (SNV), messenger RNA (mRNA) expression, and the survival landscape. Subsequently, we used the R language to generate heat maps of these genes in the three RCC subtypes and explored the correlation between these molecules. In particular, the fatty acid metabolism genes were used to establish three risk signatures related to patient prognosis in the three RCC subtypes. The findings should be valuable information for future scientific research and clinical diagnosis and treatment of RCC.

## 2. Materials and Methods

### 2.1. Data Acquisition

The Cancer Genome Atlas (TCGA) program is sponsored by the National Cancer Institute and National Human Genome Research Institute and was jointly launched in 2006. TCGA uses a large-scale gene sequence analysis technology to build a complete set of maps related to all cancer genome changes. In February 2020, we downloaded the CNV, SNV, and mRNA expression data of the KIRC, KIRP, and KICH datasets in the TCGA database. Clinical parameters and survival data of the corresponding cases were extracted. The KIRC database contains 539 tumor tissues and 72 normal tissues. The KIRP database contains 289 tumor tissues and 32 normal tissues. The KICH database contains 65 tumor tissues and 24 normal tissues.

### 2.2. GSVA

GSVA is an open-source software package for R [10]. GSVA can sensitively detect subtle pathway activity changes in samples and can be used to build path-centric biological models. We used this data packet for the three RCC subtypes.

### 2.3. Protein-Protein Interaction (PPI) Network Analysis

Genes related to fatty acid metabolism were identified through the GSEA website [16, 17]. STRING is a high-coverage, high-quality PPI network platform with a wide range of applications in interpreting large-scale biomedical data and visualization in the context of systems biology [18]. The STRING online database was used to map these fatty acid metabolism genes on the PPI network. Cytoscape visualization software was used to draw the PPI network [19].

### 2.4. Data Processing and Analysis

The R language has powerful data analysis processing and visual drawing functions. It can be used on Windows, Linux, and Mac systems. Writing a new code or adjusting an existing code can quickly achieve the requirements of data presentation and graphic drawing in scientific research. The R language was used to draw a heat map of the path changes in KIRC, KIRP, and KICH, where the defining criteria were *P* < 0.05 and logFC > 0.2. We then plotted Venn diagrams of the upregulated and downregulated pathways between the three subtypes of RCC and found pathways common to all three. The R language was additionally used to map the activation or inhibition of genes related to fatty acid metabolism pathways in KIRC, KIRP, and KICH. We plotted the heat maps of CNV, SNV, mRNA expression, and the survival landscape of fatty acid metabolism genes in these three RCC subtypes. Next, to reveal the changes in mRNA levels more clearly, the R language was used to draw a heat map of the expression of these fatty acid metabolic genes in KIRC, KIRP, and KICH. Univariate Cox analysis of these genes was performed in the three RCC subtypes to explore the gene correlations. Finally, these fatty acid metabolism genes were used to establish risk signatures related to prognosis. Multiple data packages were used coordinately. The limma software package performed different analyses of the data. The corrplot software package performed the coexpression analysis. The pheatmap software package was used to construct heat maps. The survival software package was used to analyze and construct survival curves. The survivalROC software package was used to explain and illustrate the receiver operating characteristic (ROC) curve. In addition, in order to verify the results that we obtained by analyzing the TCGA database, we used the data in the GEO database to verify the expression levels of fatty acid metabolism-related genes in KIRC, KIRP, and KICH and drew the corresponding heat map (Supplementary materials Fig. S1A-C). Among them, the GEO chip number of KIRC is GSE11151 and the GEO chip number of KIRP and KICH is GSE15641 [20–23]. Because chip GSE15641 contains both KIRP data and KICH data, therefore, we will select the KIRP and KICH data contained in this chip for subsequent verification. A *P* value < 0.05 denoted statistical significance.

## 3. Results

### 3.1. A Panoramic View of GSVA in Pan-RCC

**Figure 1:**
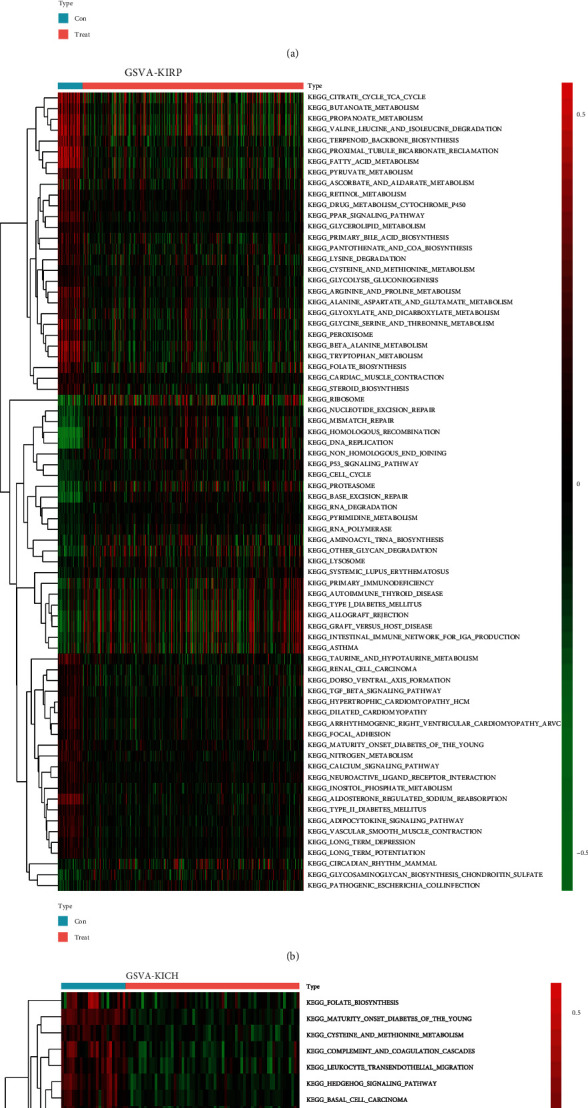
Overview of GSVA in pan-renal cell carcinoma. (a) KIRC, (b) KIRP, and (c) KICH. The redder the color, the stronger the activation of the corresponding pathway. The greener the color, the stronger the suppression of the relevant pathway.

The R language was first used to perform GSVA on pan-RCC and the corresponding heat map was generated. In KIRC, the pathways that were inhibited included folate biosynthesis, oxidative phosphorylation, steroid biosynthesis, citrate cycle, tricarboxylic acid (TCA) cycle, and fatty acid metabolism. Activated pathways included apoptosis, DNA replication, cell cycle, Notch signaling pathway, JAK/STAT signaling pathway, and P53 signaling pathway (Figure 1(a)). In KIRP, the inhibited pathways included citrate cycle, TCA cycle, fatty acid metabolism, peroxisome proliferator-activated receptor signaling pathway, transforming growth factor-beta (TGF-*β*) signaling pathway, calcium signaling pathway, and adipocytokine signaling pathway. Activated pathways included nucleotide excision repair, DNA replication, P53 signaling pathway, cell cycle, proteasome, and RNA degradation (Figure 1(b)). In KICH, the inhibited pathways included folate biosynthesis, hedgehog signaling pathway, tight junction, fatty acid metabolism, and drug metabolism cytochrome p450. Activated pathways included oxidative phosphorylation, citrate cycle, TCA cycle, protein export, RNA degradation, and mammalian target of rapamycin signaling pathway (Figure 1(c)).

### 3.2. Panoramic View of Genes Related to Fatty Acid Metabolism Pathways in RCC

**Figure 2:**
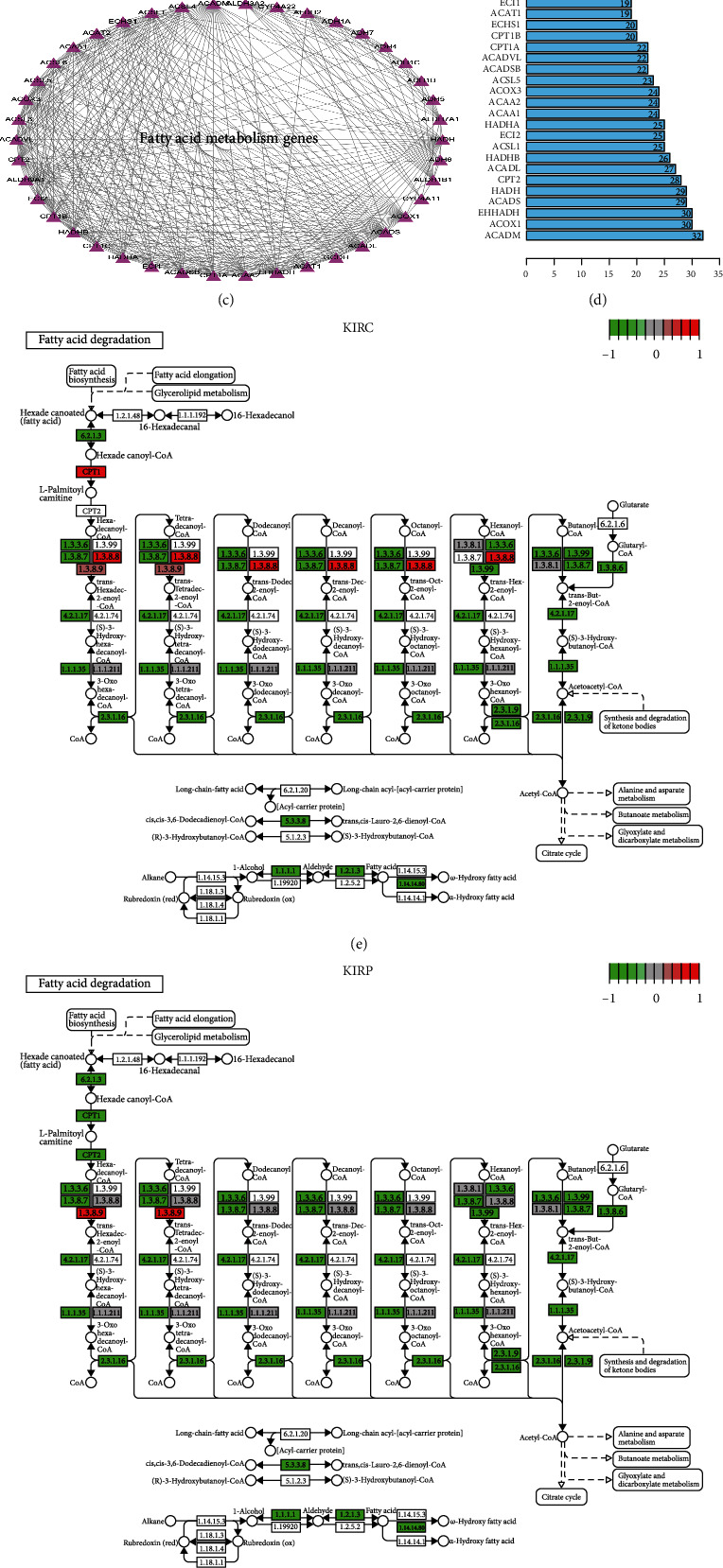
Overview of the fatty acid metabolism pathway in pan-renal cell carcinoma. (a) Venn diagrams of downregulated pathways in pan-renal cell carcinoma. (b) Venn diagrams of upregulated pathways in pan-renal cell carcinoma. (c) Interaction diagram between fatty acid metabolism genes. (d) The weight of each fatty acid metabolism gene in all the biological processes. The larger the value, the more critical is its biological role. (e) Inhibition or activation of related biological processes on fatty acid metabolism pathways in KIRC. (f) Inhibition or activation of relevant biological processes on fatty acid metabolism pathways in KIRP. (g) Inhibition or activation of related biological processes on fatty acid metabolism pathways in KICH. The redder the color, the more potent is the activation. The greener the color, the more influential is the inhibition.

Venn diagram construction identified up- and downregulated pathways in the three RCC subtypes. The homologous recombination and RNA polymerase pathways were upregulated (Figure 2(a)). The cysteine and methionine metabolism, primary bile acid biosynthesis, glycine serine and threonine metabolism, folate biosynthesis, taurine and hypotaurine metabolism, arginine and proline metabolism, butanoate metabolism, tryptophan metabolism, beta-alanine metabolism, proximal tubule bicarbonate reclamation, and fatty acid metabolism pathways were downregulated (Figure 2(b)). Fatty acid metabolism has an essential role in RCC. We identified genes related to fatty acid metabolism using the GSEA website. These genes were used to draw a PPI network map and quantify the data (Figures 2(c) and 2(d)). The findings revealed potentially critical roles of the acyl-CoA dehydrogenase medium chain (ACADM), acyl-CoA oxidase 1 (ACOX1), and enoyl-CoA hydratase and 3-hydroxyacyl-CoA dehydrogenase (EHHADH) genes in the biological process. We then assessed the expression of the fatty acid metabolism genes in three RCC subtypes and plotted a panoramic view. The plot revealed significant differences in the expression of these genes in the different RCC subtypes (Figures 2(e)–2(g)). The data highlighted the heterogeneity between the genes.

### 3.3. Molecular Changes in Fatty Acid Metabolism Genes in Pan-RCC

**Figure 3:**
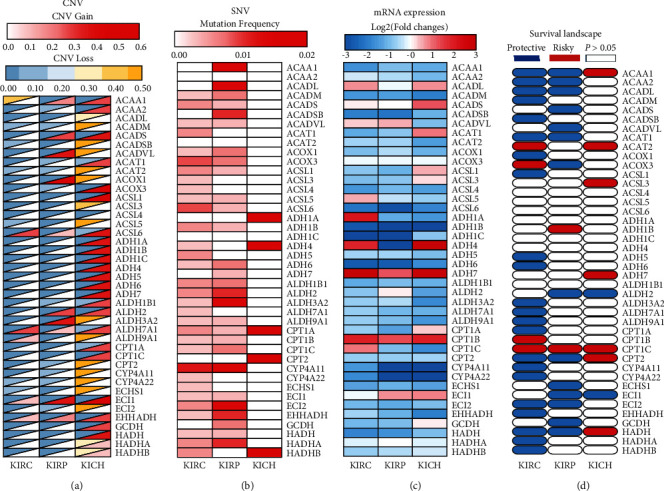
Overview of fatty acid metabolism genes in pan-renal cell carcinoma. (a) CNV in pan-renal cell carcinoma. The lower half of the rectangle represents the CNV gain. The redder the color, the higher the frequency of variation. The upper part represents the CNV loss. The yellower the color, the higher the frequency of variation. (b) SNV in pan-renal cell carcinoma. The redder the color, the higher the frequency of variation. (c) The mRNA expression of fatty acid metabolism genes in pan-renal cell carcinoma. Red indicates that the corresponding gene is upregulated in the tumor tissue. Blue indicates that the corresponding gene is downregulated in the tumor tissue. (d) The survival landscape of fatty acid metabolism genes in pan-renal cell carcinoma. Blue represents protective factors, red represents risk factors, and white represents a *P* value > 0.05, which is not statistically significant.

The R language was used to generate CNV, SNV, mRNA expression, and survival landscape panoramas of the fatty acid metabolism genes in the three RCC subtypes. The survival landscape was drawn by TBtools (http://cj-chen.github.io/tbtools/). In CNV, the related genes in KICH displayed higher acquired and deletion mutation frequencies than KIRC and KIRP (Figure 3(a)). In SNV, related genes displayed higher mutation frequencies in KIRP than KIRC and KICH (Figure 3(b)). Concerning subsequent mRNA expression, ADH7 and CPT1B were upregulated in all three RCC subtypes. In contrast, ADH1B, CYP4A11, and CYP4A22 are downregulated (Figure 3(c)). Finally, in the survival landscape panorama, blue denoted a protective factor and red denoted a risk factor. Many fatty acid metabolism genes have a protective role in KIRC. The vast majority of statistically significant genes were risk factors in KICH (Figure 3(d)). Interestingly, ACCA1 and HADH were protective factors in KIRC and KIRP but were risk factors in KICH.

### 3.4. Clinical Relevance of Fatty Acid Metabolism Genes in Pan-RCC

**Figure 4:**
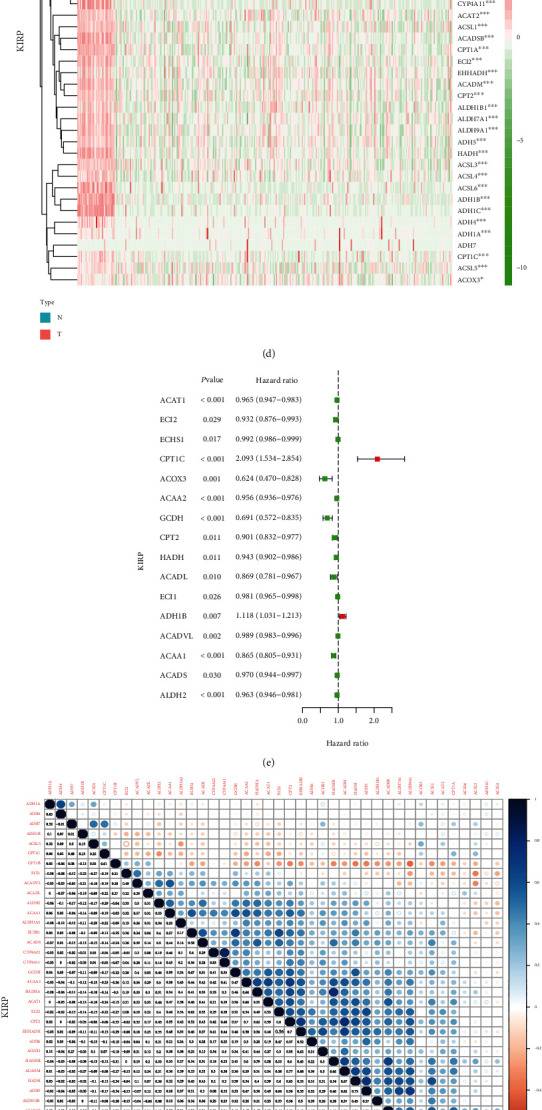
Correlation between two fatty acid metabolism genes in pan-renal cell carcinoma. (a, d, g) Expression of fatty acid metabolism-related genes between cancer tissues and normal tissues in KIRC, KIRP, and KICH. The redder the color, the higher the expression. The greener the color, the lower the expression. (b, e, h) Univariate analysis of fatty acid metabolism-related genes in KIRC, KIRP, and KICH. When the hazard ratio of this gene is >1, it means that the gene is a risk factor in the corresponding tumor and vice versa. (c, f, i) Correlation analysis of fatty acid metabolism-related genes in KIRC, KIRP, and KICH. Red represents a positive correlation and blue represents a negative correlation. ^∗^*P* < 0.05, ^∗∗^*P* < 0.01, and ^∗∗∗^*P* < 0.001.

To understand whether these genes were protective or a risk factor in tumorigenesis and development, the R language was used to generate heat maps of the expression of these fatty acid metabolic genes in the three RCC subtypes (Figures 4(a), 4(d), and 4(g)). In the figure, progressively redder and greener color indicated progressively higher and lower expression levels, respectively. To verify these results, we chose to use the data in the GEO database to verify our results. In the GEO database, we selected chips corresponding to KIRC, KIRP, and KICH. Among them, KIRC's GEO chip number is GSE11151 and KIRP and KICH's GEO chip number is GSE15641 [20–23]. Because chip GSE15641 contains both KIRP data and KICH data, therefore, we will select the KIRP and KICH data contained in this chip for subsequent verification. We use the information on these GEO chips to explore the expression of fatty acid metabolism-related genes in KIRC, KIRP, and KICH and draw the corresponding heat maps (Supplementary materials Fig. S1A-C). Subsequently, we carefully compared the expression of these fatty acid metabolism-related genes in the GEO database with the expression in the TCGA database and found that the results of the two databases are basically consistent with each other. And then, univariate Cox regression analysis of these fatty acid metabolic genes was performed in the three RCC subtypes. The hazard ratios of ACOX3, CPT1B, and CPT1C in KIRC exceeded 1, implicating the genes as risk factors in the development of KIRC (Figure 4(b)). Similarly, CPT1C and ADH1B were risk factors in the occurrence and development of KIRP (Figure 4(e)). CPT1C, ACSL3, ACAA1, CPT2, HADH, and ACAT2 were identified as risk factors in the development of KICH (Figure 4(h)). To understand the correlation between these genes, we drew a panoramic view of the relationship between the two molecules. In the upper right of these panoramic pictures, progressively bluer colors and progressively larger bubble sizes indicated increasingly stronger positive correlations between the two molecules. Progressively redder colors and progressively larger bubble sizes indicated stronger negative relationships between the two. The bottom left of these panoramas displays a quantitative value of the correlation. A value closer to 1 and −1 indicated a progressively stronger positive correlation and negative correlation, respectively. Strong positive correlations were evident between ADH4 and ADH1C in KIRC (Figure 4(c)), between HADHA and HADHB in KIRP (Figure 4(f)) and between ADH6 and ADH1A in KICH (Figure 4(i)).

### 3.5. The Prognostic Risk Signature in KIRC

**Figure 5:**
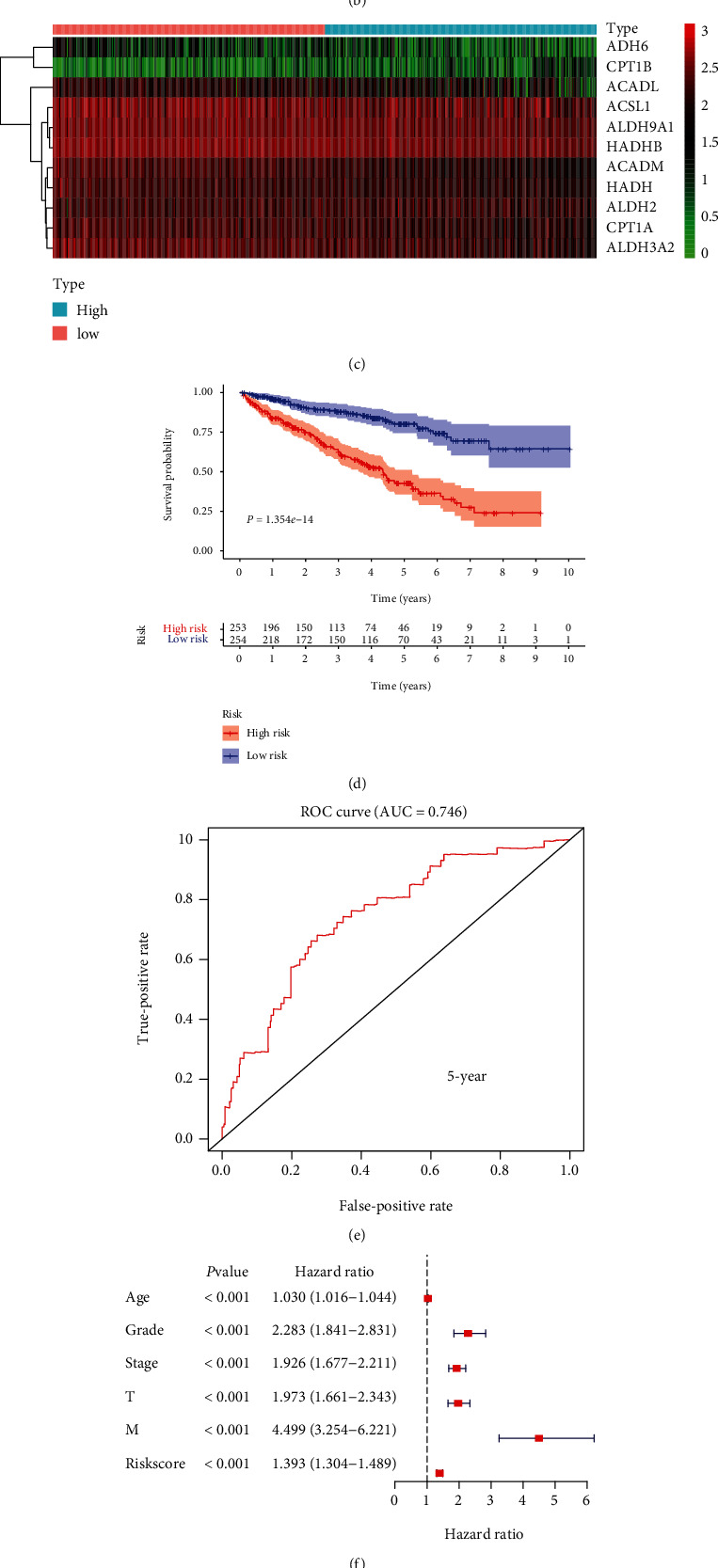
Establishment of the risk signature in KIRC and its correlation with clinical characteristics. (a–c) The process of building the risk signature containing 11 genes in KIRC. (d) Survival curve drawn based on the model. (e) Five-year ROC curve. Results of (f) univariate Cox analysis and (g) multivariate Cox analysis.

In order to explore the potential clinical application value of fatty acid metabolism genes in KIRC, we use fatty acid metabolism genes to establish a prognostic-related risk signature in KIRC (Figures 5(a) and 5(b)). This risk signature consisted of ADH6, CPT1B, ACADL, ACSL1, ALDH9A1, HADHB, ACADM, HADH, ALDH2, CPT1A, and ALDH3A2 (Figure 5(c)). This risk signature was used to divide KIRC patients into high-risk and low-risk groups. A statistically significant difference was evident between the risk signature and overall survival (*P* = 1.354*e* − 14; Figure 5(d)). An ROC curve was drawn. The area under the ROC curve (AUC) was 0.746 (Figure 5(e)). Subsequently, we used RT-qPCR to detect the mRNA expression of the ADH6 gene in three KIRC tumor samples and three normal kidney samples and plotted relative histograms (Supplementary materials Fig. S2). The results showed that the expression level of the ADH6 gene in KIRC tumor tissue was significantly lower than that in normal kidney tissue. Finally, univariate and multivariate Cox regression analyses were performed. The age, grade, stage, and risk score were independent risk factors in KIRC (Figure 5(f) and 5(g)).

### 3.6. The Prognostic Risk Signature in KIRP

**Figure 6:**
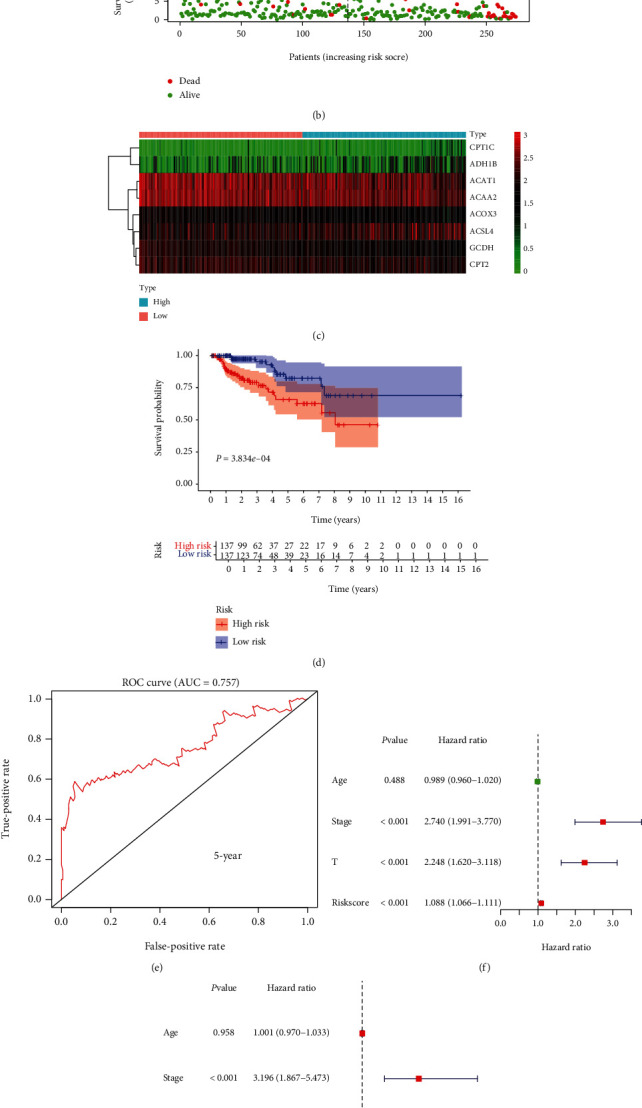
Risk signature in KIRP and its correlation with clinical characteristics. (a–c) The process of building the risk signature containing eight genes in KIRP. (d) Survival curve drawn based on the model. (e) Five-year ROC curve. Results for (f) univariate Cox analysis and (g) multivariate Cox analysis.

Similarly, a prognostic risk signature was established in KIRP (Figures 6(a) and 6(b)). This risk signature consisted of CPT1C, ADH1B, ACAT1, ACAA2, ACOX3, ACSL4, GCDH, and CPT2 (Figure 6(c)). The allocation of KIRP patients into high- and low-risk groups also proved to be statistically significant with overall survival (*P* = 3.834*e* − 04; Figure 6(d)). The AUC of the ROC curve was 0.757 (Figure 6(e)). The univariate and multivariate Cox regression analyses revealed that the stage and risk score were independent risk factors in KIRP (Figures 6(f) and 6(g)).

### 3.7. The Prognostic Risk Signature in KICH

**Figure 7:**
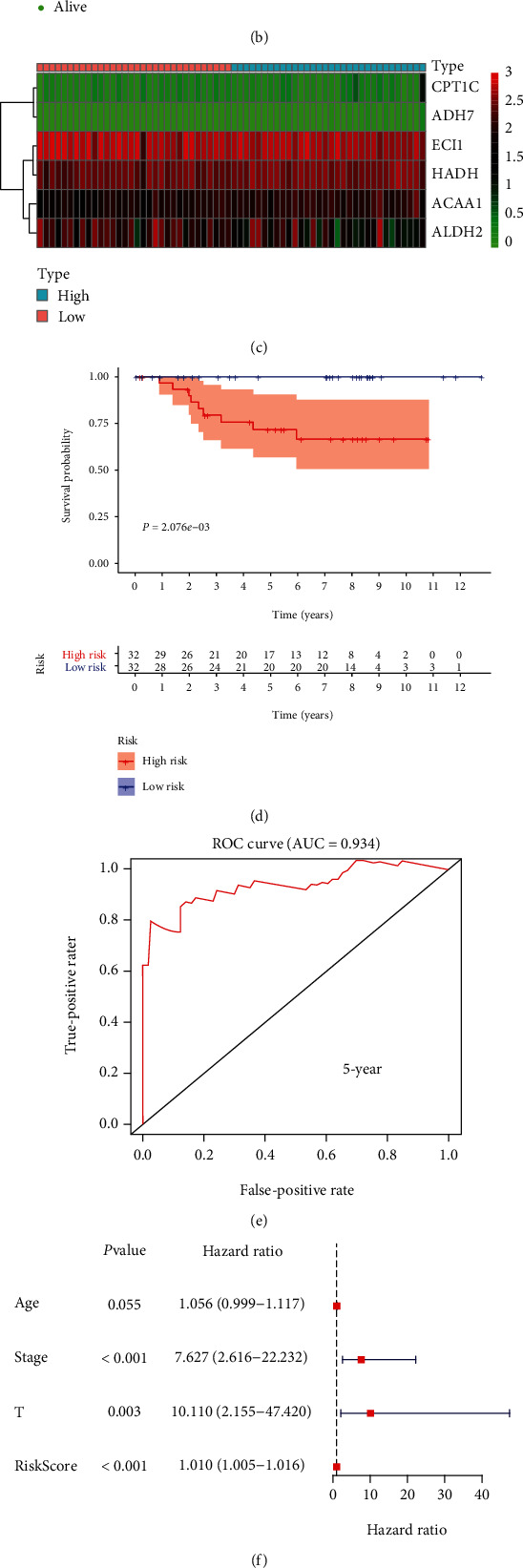
Risk signature in KICH and its correlation with clinical characteristics. (a–c) The process of building the risk signature containing six genes in KICH. (d) Survival curve drawn based on the model. (e) Five-year ROC curve. Results of (f) univariate Cox analysis and (g) multivariate Cox analysis.

The similarly constructed prognostic-related risk signature in KICH (Figures 7(a) and 7(b)) consisted of CPT1C, ADH7, ECI1, HADH, ACAA1, and ALDH2 (Figure 7(c)). The allocation of KICH patients into high- and low-risk groups also was statistically significant with overall survival (*P* = 2.076*e* − 03; Figure 7(d)). Then, AUC of the ROC curve was 0.934 (Figure 7(e)). Finally, univariate and multivariate Cox regression analyses determined that the risk score was an independent risk factor in KICH (Figures 7(f) and 7(g)).

## 4. Discussion

RCC is a common malignant tumor in the urinary system. RCC accounts for approximately 3% of all adult cancer patients [24, 25]. Once RCC has metastasized, the five-year survival rate is only 12% and approximately 20-40% of patients with primary kidney cancer experience distant metastases [26, 27]. Understanding the molecular mechanisms of the development and progression of RCC is increasingly important, and detailed and comprehensive data on the three RCC subtypes are urgently needed. Big data analysis by a high-throughput sequencing technology has identified potential diagnostic and therapeutic targets in disease progression [28, 29].

This study analyzed TCGA sequence data to discover effective prognostic models of pan-RCC. The findings could potentially guide future clinical and basic medical research. First, GSVA was used to analyze the three RCC subtypes. This analysis revealed the joint downregulation of fatty acid metabolism pathways in the three subtypes. The finding highlighted the important role of fatty acid metabolism in the three RCC subtypes. Tumor cells usually use aerobic glycolysis (Warburg effect) to meet their energy and membrane structure needs. These are precisely the key factors that drive cancer growth, immune escape, survival, and disease development. These glycolysis products are used to synthesize lipids and provide a material basis for cell proliferation. Targeted fatty acid metabolism pathways can play a substantial role in RCC [12, 30, 31]. Focusing on the fatty acid metabolism pathways, we explored the genetic variation and clinical relevance in the three RCC subtypes.

In univariate Cox regression, ACOX3, CPT1B, CPT1C, and five clinical characteristics (age, grade, stage, T, and M) were significant predictors of survival in KIRC patients. Multivariate Cox regression suggested that three clinical characteristics (age, grade, and stage) were independent prognostic factors for KIRC. Similarly, in KIRP, univariate Cox regression showed that CPT1C, ADH1B, and two clinical characteristics (stage and T) were significant predictors of survival. Multivariate Cox regression suggested that the clinical stage was an independent prognostic factor. Univariate Cox regression in KICH showed that CPT1C, ACSL3, ACAA1, CPT2, HADH, ACAT2 and two clinical characteristics (stage and T) were significant predictors of survival. However, the multivariate Cox regression did not reveal any independent prognostic factors. The collective findings revealed that the three RCC subtypes have common and different risk factors. Thus, while the subtypes are all RCC, they are heterogeneous. CPT1C was identified as having a risk factor role in all three RCC subtypes. CPT1C is the last member of the CPT1 family to be identified. The protein is mainly expressed in the endoplasmic reticulum of cells and can interact with different proteins to produce a wide range of biological effects. The most important is the interaction with Atlasin-1, which maintains the endoplasmic reticulum integrity of sex-related proteins. There is increasing evidence for the role of CPT1C in regulating lipid metabolism. The protein is highly expressed in specific tumor cells, which confers resistance to low glucose and hypoxia [32, 33]. Therefore, CPT1C may be a promising target for the treatment of cancer [34].

Finally, we used the fatty acid metabolism genes to establish prognostic risk signatures in the three RCC subtypes. The risk signature constructed in KIRC patients consists of eleven genes: ADH6, CPT1B, ACADL, ACSL1, ALDH9A1, HADHB, ACADM, HADH, ALDH2, CPT1A, and ALDH3A2. The risk signature constructed in KIRP patients consists of eight genes: CPT1C, ADH1B, ACAT1, ACAA2, ACOX3, ACSL4, GCDH, and CPT2. The risk signature created in KICH patients consists of six genes: CPT1C, ADH7, ECI1, HADH, ACAA1, and ALDH2. The possible underlying mechanisms of ADH6 in pancreatic cancer have been discovered in previous studies. These include fatty acid metabolism, retinol metabolism, primary alcohol metabolic processes, and drug metabolism cytochrome P450 [35]. Fatty acid oxidation may affect tumor progression by affecting lymphangiogenesis, and CPT1B may participate [36]. Interest in ACADL in tumor biology has focused mainly on prostate cancer, breast cancer, and esophageal squamous cell carcinoma, primarily to study its influence on the occurrence, development, and treatment of tumors. However, many potential functions are still unclear and require further research [37–39]. Studies in hepatocellular carcinoma have shown that long-chain noncoding RNA HULC can activate ACSL1 by upregulating the transcription factor PPARA to affect the proliferation of liver cancer cells [40]. Mutations in the ALDH9A1 gene may be a potential risk factor in RCC [41]. The ACADM gene can be regulated by microRNA-224 to affect the apoptosis of breast epithelial cells through the production of triglycerides [42]. After the downregulation of HADH, *β*-oxidation was inhibited in gastric cancer cells, which led to the accumulation of fatty acids. This inhibited the transcription of phosphatase and tensin homolog and promoted the proliferation and invasion of gastric cancer cells [43–45]. ALDH2 is related to the occurrence and development of liver cancer, gastric cancer, and colon cancer [46]. As a critical rate-limiting enzyme for fatty acid oxidation, CPT1A plays a role in transporting fatty acids into the mitochondria for oxidative phosphorylation. CPT1A has also been associated with the occurrence and very early development of various tumors [47–50]. ADH1B mutations have also been extensively studied and have been associated with esophageal, head and neck, ovarian, and colorectal cancer [51–54]. Acsl4 may induce ferroptosis by altering the lipid composition [55]. From previous research, it is clear that we can see that the vast majority of genes used to establish risk signatures are related to cancer research and to some extent support our findings.

In many cancers, gene expression signatures and prognostic-related risk signatures have proven to function based on their roles in driving pathogenesis, which is useful for predicting the clinical outcome and prognostic value [56–60]. The multiple models that we constructed using genes related to fatty acid metabolism can effectively predict the survival of kidney cancer patients. Even so, data from large-scale, multicenter, evidence-based medical studies are needed for verification.

## 5. Conclusions

In summary, we used TCGA data to draw a panoramic view of CNV, SNV, mRNA expression, and survival landscape of fatty acid metabolism genes in KIRC, KIRP, and KICH patients. Based on the fatty acid metabolism genes, we identified a variety of prognostic risk signatures for KIRC, KIRP, and KICH. But it must be admitted that there are still some shortcomings in this research. There is a lack of exploring the functions and molecular mechanisms of these new mRNAs in vivo and in vitro. In the future, we will continue to explore in depth along these important clues. We believe that the current data can provide help for future scientific research and the clinical diagnosis and treatment of RCC.

## Data Availability

The data used to support the findings of this study are available from the corresponding author upon request.

## Abbreviations

RCC: Renal cell carcinoma
GSVA: Gene set variation analysis
KEGG: Kyoto encyclopedia of genes and genomes
CNV: Copy number variation
SNV: Single-nucleotide variation
KIRC: Kidney renal clear cell carcinoma
KIRP: Kidney renal papillary cell carcinoma
KICH: Kidney chromophobe
TCGA: The Cancer Genome Atlas
ADH6: Alcohol dehydrogenase 6
CPT1B: Carnitine palmitoyltransferase 1B
ACADL: Acyl-CoA dehydrogenase long chain
ACSL1: Acyl-CoA synthetase long-chain family member 1
ALDH9A1: Aldehyde dehydrogenase 9 family member A1
HADHB: Hydroxyacyl-CoA dehydrogenase trifunctional multienzyme complex subunit beta
ACADM: Acyl-CoA dehydrogenase medium chain
HADH: Hydroxyacyl-CoA dehydrogenase
ALDH2: Aldehyde dehydrogenase 2
CPT1A: Carnitine palmitoyltransferase 1A
ALDH3A2: Aldehyde dehydrogenase 3 family member A2
CPT1C: Carnitine palmitoyltransferase 1C
ADH1B: Alcohol dehydrogenase 1B
ACAT1: Acetyl-CoA acetyltransferase 1
ACAA2: Acetyl-CoA acyltransferase 2
ACOX3: Acyl-CoA oxidase 3
ACSL4: Acyl-CoA synthetase long-chain family member 4
GCDH: Glutaryl-CoA dehydrogenase
CPT2: Carnitine palmitoyltransferase 2
ADH7: Alcohol dehydrogenase 7
ECI1: Enoyl-CoA delta isomerase 1
ACAA1: Acetyl-CoA acyltransferase 1.

## Supplementary Materials

Supplementary Materials Figure S1: verification process from GEO database; Figure S2: RT-qPCR technology verifies the expression of the ADH6 gene in KIRC tumors and normal tissues.

## References

[1] Capitanio U., Montorsi F. (2016). Renal cancer. *Lancet*.

[2] Znaor A., Lortet-Tieulent J., Laversanne M., Jemal A., Bray F. (2015). International variations and trends in renal cell carcinoma incidence and mortality. *European Urology*.

[3] Sanchez D. J., Simon M. C. (2018). Genetic and metabolic hallmarks of clear cell renal cell carcinoma. *Biochimica Et Biophysica Acta. Reviews on Cancer*.

[4] Guo K., Chen Q., He X. (2018). Expression and significance of cystatin-C in clear cell renal cell carcinoma. *Biomedicine & Pharmacotherapy*.

[5] The Cancer Genome Atlas Research Network (2013). Comprehensive molecular characterization of clear cell renal cell carcinoma. *Nature*.

[6] Gatto F., Nookaew I., Nielsen J. (2014). Chromosome 3p loss of heterozygosity is associated with a unique metabolic network in clear cell renal carcinoma. *Proceedings of the National Academy of Sciences of the United States of America*.

[7] Hakimi A. A., Reznik E., Lee C. H. (2016). An integrated metabolic atlas of clear cell renal cell carcinoma. *Cancer Cell*.

[8] Morris M. R., Latif F. (2017). The epigenetic landscape of renal cancer. *Nature Reviews. Nephrology*.

[9] Schmidt L. S., Linehan W. M. (2016). Genetic predisposition to kidney cancer. *Seminars in Oncology*.

[10] Hänzelmann S., Castelo R., Guinney J. (2013). GSVA: gene set variation analysis for microarray and RNA-seq data. *BMC Bioinformatics*.

[11] Wu G., Wang Q., Xu Y. (2019). Targeting the transcription factor receptor LXR to treat clear cell renal cell carcinoma: agonist or inverse agonist?. *Cell Death & Disease*.

[12] Wu G., Xu Y., Wang Q. (2019). FABP5 is correlated with poor prognosis and promotes tumour cell growth and metastasis in clear cell renal cell carcinoma. *European Journal of Pharmacology*.

[13] Röhrig F., Schulze A. (2016). The multifaceted roles of fatty acid synthesis in cancer. *Nature Reviews. Cancer*.

[14] Vriens K., Christen S., Parik S. (2019). Evidence for an alternative fatty acid desaturation pathway increasing cancer plasticity. *Nature*.

[15] Koundouros N., Poulogiannis G. (2020). Reprogramming of fatty acid metabolism in cancer. *British Journal of Cancer*.

[16] Mootha V. K., Lindgren C. M., Eriksson K. F. (2003). PGC-1alpha-responsive genes involved in oxidative phosphorylation are coordinately downregulated in human diabetes. *Nature Genetics*.

[17] Subramanian A., Tamayo P., Mootha V. K. (2005). Gene set enrichment analysis: a knowledge-based approach for interpreting genome-wide expression profiles. *Proceedings of the National Academy of Sciences of the United States of America*.

[18] Szklarczyk D., Gable A. L., Lyon D. (2019). STRING v11: protein-protein association networks with increased coverage, supporting functional discovery in genome-wide experimental datasets. *Nucleic Acids Research*.

[19] Shannon P., Markiel A., Ozier O. (2003). Cytoscape: a software environment for integrated models of biomolecular interaction networks. *Genome Research*.

[20] Jones J., Otu H., Spentzos D. (2005). Gene signatures of progression and metastasis in renal cell cancer. *Clinical Cancer Research*.

[21] Yusenko M. V., Kuiper R. P., Boethe T., Ljungberg B., van Kessel A. G., Kovacs G. (2009). High-resolution DNA copy number and gene expression analyses distinguish chromophobe renal cell carcinomas and renal oncocytomas. *BMC Cancer*.

[22] Yusenko M. V., Zubakov D., Kovacs G. (2009). Gene expression profiling of chromophobe renal cell carcinomas and renal oncocytomas by affymetrix genechip using pooled and individual tumours. *International Journal of Biological Sciences*.

[23] Yusenko M. V., Ruppert T., Kovacs G. (2010). Analysis of differentially expressed mitochondrial proteins in chromophobe renal cell carcinomas and renal oncocytomas by 2-D gel electrophoresis. *International Journal of Biological Sciences*.

[24] Del Vecchio S., Ellis R. J. (2018). Cabozantinib for the management of metastatic clear cell renal cell carcinoma. *Journal of Kidney Cancer and VHL*.

[25] Majer W., Kluzek K., Bluyssen H., Wesoły J. (2015). Potential approaches and recent advances in biomarker discovery in clear-cell renal cell carcinoma. *Journal of Cancer*.

[26] Atkins M. B., Tannir N. M. (2018). Current and emerging therapies for first-line treatment of metastatic clear cell renal cell carcinoma. *Cancer Treatment Reviews*.

[27] Cao Q., Ruan H., Wang K. (2018). Overexpression of PLIN2 is a prognostic marker and attenuates tumor progression in clear cell renal cell carcinoma. *International Journal of Oncology*.

[28] Xu Y., Wu G., Li J. (2020). Screening and identification of key biomarkers for bladder cancer: a study based on TCGA and GEO data. *BioMed Research International*.

[29] Wu G., Zhang Z., Tang Q. (2019). Study of FABP’s interactome and detecting new molecular targets in clear cell renal cell carcinoma. *Journal of Cellular Physiology*.

[30] Courtney K. D., Bezwada D., Mashimo T. (2018). Isotope Tracing of Human Clear Cell Renal Cell Carcinomas Demonstrates Suppressed Glucose Oxidation _In Vivo_. *Cell Metabolism*.

[31] Fritz V., Benfodda Z., Rodier G. (2010). Abrogation of de novo lipogenesis by stearoyl-CoA desaturase 1 inhibition interferes with oncogenic signaling and blocks prostate cancer progression in mice. *Molecular Cancer Therapeutics*.

[32] Zaugg K., Yao Y., Reilly P. T. (2011). Carnitine palmitoyltransferase 1C promotes cell survival and tumor growth under conditions of metabolic stress. *Genes & Development*.

[33] Reilly P. T., Mak T. W. (2012). Molecular pathways: tumor cells co-opt the brain-specific metabolism gene CPT1C to promote survival. *Clinical Cancer Research*.

[34] Casals N., Zammit V., Herrero L., Fadó R., Rodríguez-Rodríguez R., Serra D. (2016). Carnitine palmitoyltransferase 1C: From cognition to cancer. *Progress in Lipid Research*.

[35] Liao X., Huang R., Liu X. (2017). Distinct prognostic values of alcohol dehydrogenase mRNA expression in pancreatic adenocarcinoma. *Oncotargets and Therapy*.

[36] Wong B. W., Wang X., Zecchin A. (2017). The role of fatty acid *β*-oxidation in lymphangiogenesis. *Nature*.

[37] Xie B. X., Zhang H., Wang J. (2011). Analysis of differentially expressed genes in LNCaP prostate cancer progression model. *Journal of Andrology*.

[38] Li Z., Heng J., Yan J. (2016). Integrated analysis of gene expression and methylation profiles of 48 candidate genes in breast cancer patients. *Breast Cancer Research and Treatment*.

[39] Yu D. L., Li H. W., Wang Y. (2018). Acyl-CoA dehydrogenase long chain expression is associated with esophageal squamous cell carcinoma progression and poor prognosis. *Oncotargets and Therapy*.

[40] Cui M., Xiao Z., Wang Y. (2015). Long noncoding RNA HULC modulates abnormal lipid metabolism in hepatoma cells through an miR-9-mediated RXRA signaling pathway. *Cancer Research*.

[41] Henrion M. Y. R., Purdue M. P., Scelo G. (2015). Common variation at 1q24.1 (ALDH9A1) is a potential risk factor for renal cancer. *PLoS One*.

[42] Shen B., Pan Q., Yang Y. (2017). miR-224 affects mammary epithelial cell apoptosis and triglyceride production by downregulatingACADMandALDH2genes. *DNA and Cell Biology*.

[43] Huang D., Li T., Li X. (2014). HIF-1-mediated suppression of acyl-CoA dehydrogenases and fatty acid oxidation is critical for cancer progression. *Cell Reports*.

[44] Yin K., Wang L., Zhang X. (2017). Netrin-1 promotes gastric cancer cell proliferation and invasion via the receptor neogenin through PI3K/AKT signaling pathway. *Oncotarget*.

[45] Shen C., Song Y. H., Xie Y. (2017). Downregulation of HADH promotes gastric cancer progression via Akt signaling pathway. *Oncotarget*.

[46] Li R., Zhao Z., Sun M., Luo J., Xiao Y. (2016). ALDH2 gene polymorphism in different types of cancers and its clinical significance. *Life Sciences*.

[47] Wang Y. N., Zeng Z. L., Lu J. (2018). CPT1A-mediated fatty acid oxidation promotes colorectal cancer cell metastasis by inhibiting anoikis. *Oncogene*.

[48] Xiong Y., Liu Z., Zhao X. (2018). CPT1A regulates breast cancer-associated lymphangiogenesis via VEGF signaling. *Biomedicine & Pharmacotherapy*.

[49] Shi J., Fu H., Jia Z., He K., Fu L., Wang W. (2016). High expression of CPT1A predicts adverse outcomes: a potential therapeutic target for acute myeloid leukemia. *eBioMedicine*.

[50] Joshi M., Stoykova G. E., Salzmann-Sullivan M. (2019). CPT1A supports castration-resistant prostate cancer in androgen-deprived conditions. *Cells*.

[51] Gharpure K. M., Lara O. D., Wen Y. (2018). ADH1B promotes mesothelial clearance and ovarian cancer infiltration. *Oncotarget*.

[52] Gu H., Gong D., Ding G. (2012). A variant allele of ADH1B and ALDH2, is associated with the risk of esophageal cancer. *Experimental and Therapeutic Medicine*.

[53] Crous-Bou M., Rennert G., Cuadras D. (2013). Polymorphisms in alcohol metabolism genes ADH1B and ALDH2, alcohol consumption and colorectal cancer. *PLoS One*.

[54] Zhang Y., Gu N., Miao L., Yuan H., Wang R., Jiang H. (2015). Alcohol dehydrogenase-1B Arg47His polymorphism is associated with head and neck cancer risk in Asian: a meta-analysis. *Tumour Biology*.

[55] Doll S., Proneth B., Tyurina Y. Y. (2017). ACSL4 dictates ferroptosis sensitivity by shaping cellular lipid composition. *Nature Chemical Biology*.

[56] Askeland E. J., Chehval V. A., Askeland R. W. (2015). Cell cycle progression score predicts metastatic progression of clear cell renal cell carcinoma after resection. *Cancer Biomarkers*.

[57] Rini B. I., Escudier B., Martini J. F. (2018). Validation of the 16-gene recurrence score in patients with locoregional, high-risk renal cell carcinoma from a phase III trial of adjuvant sunitinib. *Clinical Cancer Research*.

[58] Brooks S. A., Brannon A. R., Parker J. S. (2014). ClearCode34: a prognostic risk predictor for localized clear cell renal cell carcinoma. *European Urology*.

[59] Xu Y., Li X., Han Y. (2020). A New Prognostic Risk Model Based on PPAR Pathway-Related Genes in Kidney Renal Clear Cell Carcinoma. *PPAR Research*.

[60] Zhang Y., Chen M., Liu M., Xu Y., Wu G. (2021). Glycolysis-related genes serve as potential prognostic biomarkers in clear cell renal cell carcinoma. *Oxidative Medicine and Cellular Longevity.*.

