# Supplementary material for: Comparison of the Fatty Acid Metabolism Pathway in Pan-Renal Cell Carcinoma: Evidence from Bioinformatics
Source: Anal Cell Pathol (Amst). 2021 Feb 22;2021:8842105. doi: 10.1155/2021/8842105 (PMC7925032; doi:10.1155/2021/8842105)
Supplement: Supplementary Materials — Figure S1: verification process from GEO database; Figure S2: RT-qPCR technology verifies the expression of the ADH6 gene in KIRC tumors and normal tissues. [file 8842105.f1.docx]

**Supplementary materials**


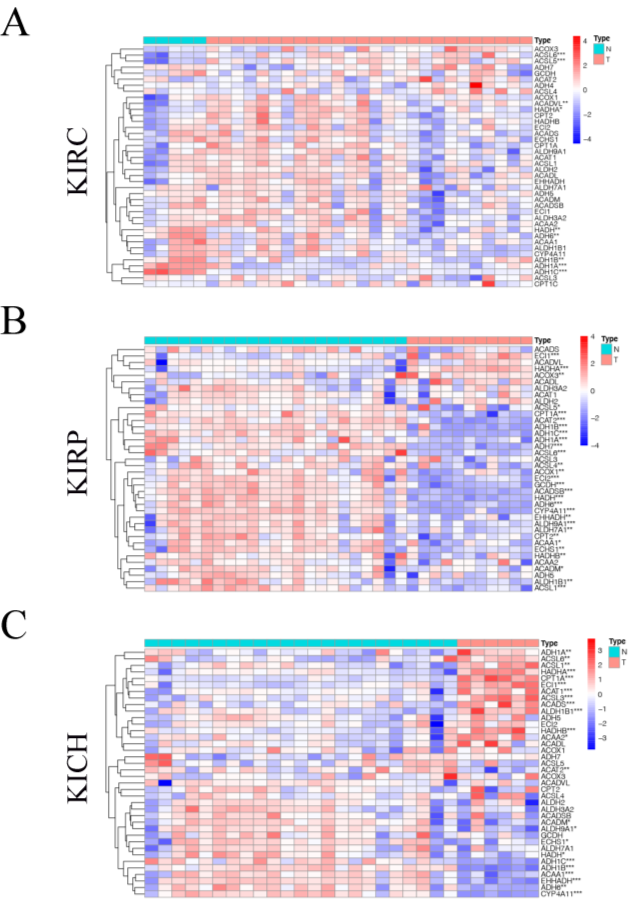


**Figure S1:** Verification process from GEO database. Heat map of the expression of fatty acid metabolism related genes in (A) KIRC, (B) KIRP and (C)KICH.

**Figure S2:** A histogram drawn based on the expression level of ADH6 gene in three pairs of KIRC tumor tissues and normal tissues. ***P < 0.001.
